# Simple and Efficient Chemically Defined In Vitro Maturation and Embryo Culture System for Bovine Embryos

**DOI:** 10.3390/ani12213057

**Published:** 2022-11-07

**Authors:** María Elena Arias, Tamara Vargas, Victor Gallardo, Luis Aguila, Ricardo Felmer

**Affiliations:** 1 Laboratory of Reproduction, Centre of Reproductive Biotechnology (CEBIOR-BIOREN), Universidad de La Frontera, Temuco 4811322, Chile; 2 Department of Agricultural Production, Faculty of Agriculture and Environmental Sciences, Universidad de La Frontera, Temuco 4811322, Chile; 3 Department of Agricultural Sciences and Natural Resources, Faculty of Agriculture and Environmental Sciences, Universidad de La Frontera, Temuco 4811322, Chile

**Keywords:** chemically defined, IVF, zygote, bovine oocyte, EGF, embryos, FGF2 and IGF1

## Abstract

**Simple Summary:**

Supplementation of the culture media for the in vitro production of bovine embryos with fetal bovine serum (FBS) is associated with inconsistent outcomes. This study replaced FBS in both maturation and embryo culture media by different growth factors. The results show that FBS is dispensable for in vitro maturation, but not gonadotropins. In addition, embryos generated under completely defined conditions (absence of FBS and BSA during oocyte maturation and embryo culture) showed similar in vitro development and survival rate post thawing compared to undefined conditions carrying FBS. Thus, we report a simple defined IVP system for bovine species that generates developmental outcomes and embryos of similar quality than those produced under conditions containing FBS and BSA.

**Abstract:**

Supplementation of the culture media for in vitro production (IVP) of bovine embryos with fetal bovine serum (FBS) is associated with inconsistent outcomes. The present study sought to replace FBS and BSA by insulin-like growth factor 1 (IGF1), fibroblast growth factor 2 (FGF2) and epidermal growth factor (EGF). In Experiment 1, absence of FBS from maturation medium (MM) did not affect the rate of in vitro maturation, as assessed by the extrusion of the first polar body. However, when gonadotropins and FBS were removed from the MM, the maturation rate was significantly reduced even in the presence of growth factors. Therefore, gonadotropin-supplemented MM medium was established as the base medium for the defined maturation condition. In Experiment 2, the addition of growth factors to gonadotropin-supplemented MM medium supported similar maturation (~90%) compared to the undefined condition (FBS-carrying). In Experiment 3, the addition of growth factors to embryo culture medium showed similar in vitro competence compared to the undefined (FBS) control. In Experiment 4, completely defined conditions (absence of FBS and BSA during in vitro maturation and embryo culture) were tested. A higher cleavage was observed with FGF2 (86%) compared to EGF (77%) and the FBS control (77%), but similar blastocyst rates were observed for FGF2 (24%), EGF (19%) and the FBS control (25%). Embryo quality was similar among groups. Finally, post-thawing survival was higher for FGF2 (94%) compared to the FBS control (77%). Thus, we report a simple defined IVP system for bovine species that generates developmental outcomes and embryos of similar quality than those produced under conditions containing FBS.

## 1. Introduction

In the last decades, bovine in vitro fertilization (IVF) has evolved from an experimental procedure for treating infertile animals to a tool for genetic selection and modification [1]. Currently with the aid of ultrasound-guided transvaginal oocyte retrieval, IVF has gained increasing interest by researchers as an alternative to multiple ovulation embryo transfer programs, particularly in South American countries [2]. Thus, it is not surprising that many researchers have put their effort seeking to improve in vitro culture conditions.

Culture media for in vitro production (IVP) of bovine embryos have been regularly supplemented with different growth factors, antioxidants, and macromolecules as a source of protein [3]. For instance, the main source of protein used to boost the developmental competence of in vitro produced embryos of non-rodent species is fetal bovine serum (FBS). However, its variable composition, due to the limited definition of their components, and the great variation of each batch or supplier [4], are the main factors associated with the lack of consistent outcomes observed among different research groups [5,6]. 

The addition of FBS to the culture medium has shown a biphasic effect on the developmental potential of the growing embryos, either inhibiting the early stages of development or stimulating morulae and blastocyst differentiation [4,7,8]. These include mitochondrial alterations [9,10], the presence of dark clusters [5,11], a high incidence of apoptosis [6], low protein synthesis [12], a lower ratio of inner cell mass: trophoblast cells [13] and changes in the expression pattern of genes important for early embryonic development [14]. In addition, it is suspected to contribute to the condition known as large offspring syndrome (LOS) [10].

Taken together, this background strongly suggests the need for a reliable serum-free culture system capable of producing results comparable to a conventional FBS-based system. Previous studies have reported the use of chemically defined serum-free culture conditions for bovine embryos through the replacement of FBS or BSA for polyvinyl pyrrolidone (PVP), polyvinyl alcohol (PVA), commercial serum replacers, or supplementing these media with different growth factors. These media include synthetic oviductal fluid (SOF) [15], CR1aa [16], bovine embryo culture medium (BECM) [17], IVD101 [18], and potassium simplex optimization medium (KSOM) [19,20]. Although these media support preimplantation embryonic development up to the blastocyst stage, their efficiency must be boosted by supplementing with FBS [21,22]. Furthermore, an efficient and economic defined IVP system in which maturation and embryo culture are carried out without FBS has not been reported in the bovine species. Because it has been recently described that insulin-like growth factor 1 (IGF1), fibroblast growth factor 2 (FGF2) and epidermal growth factor (EGF) have the potential to improve the developmental potential and quality of bovine embryos generated in vitro [23,24,25], the present study aimed to replace FBS and BSA of the culture media used during the IVP of bovine embryos by evaluating the addition of IGF1, FGF2 or EGF individually to the in vitro maturation and embryo culture media.

## 2. Materials and Methods

Unless stated otherwise, all chemicals were purchased from Sigma-Aldrich (St. Louis, MO, USA).

### 2.1. In Vitro Maturation (IVM)

Ovaries were collected from a local slaughterhouse (Frigorifico Temuco, Temuco, Chile). Cumulus oocyte complexes (COCs) were aspirated from 2 to 7 mm follicles using an 18-gauge needle. COCs were selected according to their morphology [26] and cultured in TCM-199 medium supplemented with 10% inactivated FBS (Hyclone Laboratories, Inc., Logan, UT, USA) or with growth factors and 6 µg/ml luteinising hormone (LH; Sioux Biochemical Inc., Sioux City, IA, USA), 6 µg/ml follicle-stimulating hormone (FSH; Sioux Biochemical Inc., Sioux City, IA, USA) and 1 µg/ml estradiol (E_2_). For some experiments, the maturation (TCM-base) medium was devoid of gonadotropins (LF, FSH, E_2_) and/or FBS. COCs were incubated for 22–24 h at 38.5 °C in 5% CO_2_ and saturation humidity. Maturation rate was calculated by considering the number of oocytes that extruded the first polar body in relation to total number of oocytes in culture.

### 2.2. In Vitro Fertilization (IVF)

In vitro-matured oocytes were co-incubated with 1 × 10^6^ Percoll-separated frozen–thawed sperm per mL (Alta Genetics Inc., Alberta, Canada) for 18 h at 38.5 °C and 5% CO_2_ in IVF-TL supplemented with 0.2 mM sodium pyruvate, 6 mg fatty acid-free BSA and 0.025 mg gentamicin sulphate per ml [27]. Final IVF-TALP contained 80 µM penicillamine, 40 µM hypotaurine, 10 µM epinephrine and 2 µg/ml heparin.

### 2.3. In Vitro Embryo Culture

Groups of approximately 30 presumptive zygotes were cultured in 50 µL drops under mineral oil in the following culture medium: 1) KSOM (EmbryoMax^®^, Millipore Corp, Billerica, MA, USA) supplemented with 1% (*v*/*v*) BME-essential amino acids, 1% (*v*/*v*) MEM-nonessential amino acids and 4 mg/mL fatty acid free BSA. Culture was carried out at 38.5 °C with a gas mixture of 5% CO_2_, 5% O_2_, 90% N_2_ and saturation humidity. Cleavage rate was recorded on day 3 of culture, at which time embryos in the control group were supplemented with 5% FBS (undefined conditions). Embryos were further cultured until Day 7 and/or Day 9, depending on the experiment. Hatching rate was calculated regarding the number of hatched blastocysts in relation to the total number of blastocysts. For all chemically defined treatments (see experimental design below) embryos were cultured in one step until day 7 and/or 9 under the same temperature and gas conditions as described above. For the chemically defined embryo culture medium we modified our undefined KSOM culture recipe [28] by replacing BSA by PVA and supplementing with 1.5 mM Fructose, as it had been previously described that supplementation of fructose in chemically defined protein-free medium enhances the in vitro development in bovine transgenic cloned embryos [29]. Different growth factors (EGF, IGF1 and FGF2) diluted in PBS were added to this culture medium.

### 2.4. Total Number of Cells

Morphologically expanded blastocysts (200–250 μm in diameter) were used for quality assessment [13,30]. Briefly, expanded blastocysts were fixed and washed twice in PBS/BSA and incubated for 30 min at RT in 4% paraformaldehyde containing 10 μg/ml of Hoechst. Finally, the embryos were transferred to slides on drops of 10 μl of 1:1 glycerol/PBS and examined under an epifluorescence microscope (NIKON eclipse TS100F, equipped with UV-2E/C DAPI and/or EGFP filters).

### 2.5. Terminal Deoxynucleotidyl Transferase dUTP Nick-End Labelling 

Terminal deoxynucleotidyl transferase dUTP nick-end labelling (TUNEL) was performed according to the manufacturer’s protocol using an *In Situ* Cell Death Detection Kit (Roche Diagnostics Corp.). Briefly, after fixation and permeabilization steps described above, embryos were washed twice with PVP–PBS and incubated in the dark with fluorescently conjugated terminal deoxynucleotide transferase dUTP for 1 h at 37 °C. TUNEL-stained embryos were then washed with PVP–PBS and incubated in PVP–PBS containing 10 μg/mL Hoechst 33,342 for 10 min. Embryos were mounted onto glass slides in 15 μL drops of glycerol:PBS (1:1, *v*/*v*), and visualized under an epifluorescence Eclipse TS-100F microscope (Nikon) equipped with appropriate ultraviolet filters.

### 2.6. OPS Vitrification and Warming

Vitrification was performed as previously described by Vajta et al. [31], with modifications. Day 7 expanded, and morphologically intact blastocysts were manipulated on a heated stage at 38 °C. Embryos (4–6/group) were incubated in 10% ethylene glycol (EG) and 10% dimethyl sulfoxide (DMSO) in holding medium with 10% FBS for 3 min, and then moved to a second vitrification solution of 20% EG, 20% DMSO, 0.15 M sucrose and 10% FBS. Embryos were then loaded by capillarity in a small volume into the narrow end of the OPS straw (Minitube, Valencia, Spain) and submerged immediately into liquid nitrogen for ~3 weeks until analysis. Warming was performed by immersing the end of the straw containing embryos into 38 °C holding medium plus 0.25 M sucrose for 1 min and then embryos were transferred into holding medium containing 0.15 M sucrose for another 5 min, then exposed twice (5 min each) to holding medium before the embryo culture that was carried out as described above.

### 2.7. Experimental Design 

All experiments were carried out with the same batch of oocytes distributed randomly to each treatment. Experiment 1 analyzed the effects of removing FBS and/or the gonadotropins (LH, FSH, E_2_) from the maturation medium on in vitro maturation rate. Experiment 2 evaluated the effects of replacing FBS and BSA with different growth factors in gonadotropin-containing maturation medium. Oocyte maturation was assessed by the occurrence of extrusion of the first polar body after maturation in defined conditions: TCM-199 containing FSH, LH, E_2_, and the different growth factors: 10 ng/ml epidermal growth factor (EGF), 100 ng/ml insulin-like growth factor (IGF1) or 500 ng/ml fibroblast growth factor (FGF2). Control conditions included 10% FBS (undefined conditions) as described above. Experiment 3 evaluated the effect of supplementing the embryo culture medium with different growth factors. Oocytes matured in vitro in the presence of FBS were fertilized and presumptive zygotes were randomly allocated to chemically defined KSOM-based culture medium containing 10 ng/ml EGF, or 100 ng/ml IGF1 or 500 ng/ml FGF2, 0.01% PVA and 1.5 mM fructose. Cleavage (Day 3), blastocyst rate (Day 7) and hatched blastocyst rates (Day 9) were recorded and compared to undefined culture medium as described above. Experiment 4 evaluated the developmental potential and quality of bovine embryos generated in completely defined in vitro conditions by using oocytes matured in defined maturation conditions (Experiments 1 and 2) and then culturing these oocytes in defined embryo culture conditions (Experiment 3). Cleavage (Day 3), blastocyst rate (Day 7), hatched blastocyst rate (Day7) and embryo quality (Day 7) were recorded and compared to undefined conditions. Experiment 5 evaluated the cryotolerance of embryos generated under chemically defined conditions (KSOM+FGF2) compared to undefined conditions. Re-expansion, hatching and survival rates were evaluated at 24 and 48 h after warming. Total number of cells and TUNEL positive cells were also evaluated at 48 h. Blastocysts that regained their original shape with a fully re-expanded blastocele and those that only partially regained their spherical shape were regarded as viable blastocysts, as previously described by Men et al. (2006) [31]. Then, the blastocysts were culture for additional 48 h to investigate their ability to hatch in vitro.

### 2.8. Statistical Analysis

Data were analyzed using descriptive statistics based on the mean and standard error calculated for each of the variables using Stat Graphics Plus 5.1 software (Stat Point Technologies Inc., Warrenton, VA, USA). At least 3 biological replicates were performed in each experiment. Differences among treatments were analyzed using one-way ANOVA after arcsine transformation of the proportional data. Post-hoc analysis to identify differences between groups was performed using Scheffe’s test. Differences between groups were considered significant if *p* < 0.05.

## 3. Results

### 3.1. Effects of Removing FBS and/or Gonadotropins (LH, FSH, E_2_) from the Maturation Medium on In Vitro Maturation Rates (Experiment 1)

This experiment showed that the removal of FBS from the in vitro maturation medium (TCM medium carrying hormone cocktail) does not affect the ability of COC’s to undergo nuclear maturation in vitro, as evaluated by the extrusion of the polar body (Table 1). However, when gonadotropins and FBS were removed from the maturation medium, the maturation rate was lower even in the presence of growth factors (Table 1), suggesting that gonadotropins are essentials for the induction of in vitro maturation of bovine oocytes. Therefore, gonadotropin-supplemented TCM-199 medium was established as the base medium for the defined in vitro maturation condition.

### 3.2. Effect of Supplementing EGF, IGF1 and FGF2 in Defined Maturation Medium on the Maturation Rate of Bovine Oocytes (Experiment 2)

The results of eight replicates with a total of 802 oocytes randomly in vitro matured in gonadotropin-supplemented TCM-199 indicate similar maturation outcomes (range 88–90%) between culturing containing EGF, IGF1, or FGF2 and the undefined FBS medium (Table 2). 

### 3.3. Effect of Supplementing EGF, IGF1 and FGF2 in Defined KSOM Culture Medium on In Vitro Developmental Potential of Bovine Embryos (Experiment 3)

The results of 10 replicates with a total of 1.000 IVF bovine oocytes showed no differences in the cleavage rate (range 79–85%) and blastocyst formation rates (range 25–30%) among groups (Table 3). Although the hatching rate was similar on Day 7 (data not shown), a higher hatching rate (*p* < 0.05) was recorded on Day 9 in the group of embryos cultured under defined KSOM medium supplemented with IGF1 compared to the undefined FBS group (Table 3).

### 3.4. Effect of EGF, IGF1 and FGF2 in Defined IVP System on the In Vitro Development and Embryo Quality of Bovine Embryos (Experiment 4) 

The results of 12 replicates with a total of 1.188 IVF zygotes showed differences in the cleavage rate in embryos matured and cultured in defined IVP conditions (in vitro maturation and embryo culture) containing FGF2 (86%) compared to EGF (77%) and the undefined conditions (77%) (Table 4). The blastocyst rate was lower (*p* < 0.05) in embryos cultured in defined IVP conditions containing IGF1 compared to the undefined conditions (Table 4). However, no differences were observed between the undefined conditions (FBS/BSA) and the embryos produced in defined IVP system containing FGF2 or EGF (Table 4). Quality of embryos, according to the total cell number, was similar among all groups (Table 4).

Cell counts were recorded at 168 h in expanded blastocysts (12 blastocysts for each treatment). FBS: fetal bovine serum; IGF1: insulin-like growth factor 1, FGF2: fibroblast growth factor 2; EGF: epidermal growth factor; ICM: inner cell mass; TE: trophectoderm. No differences were observed between treatments (*p* > 0.05).

### 3.5. Post-Cryopreservation Survival and Embryo Quality (Experiment 5)

**Figure 1:**
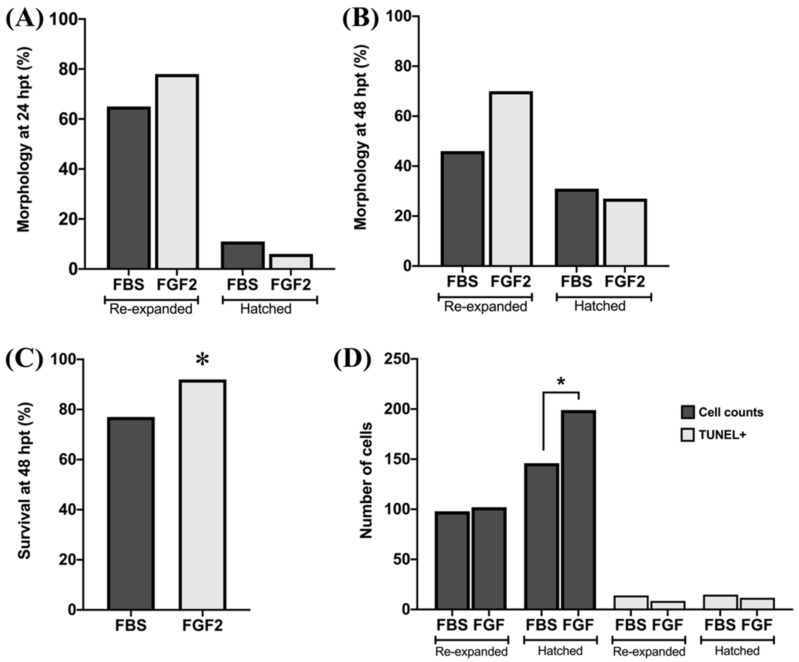
Effect of embryo culture system (defined vs. undefined) on the morphology, survival rate and cell count of vitrified embryos after warming. Embryos were analyzed at 24 h (**A**) and 48 h post thawing (**B**–**D**). FBS: fetal bovine serum; FGF2: fibroblast growth factor 2. Cell counts: total number of cells. TUNEL+: cells positives to the terminal deoxynucleotidyl transferase dUTP nick end labeling. * *p* < 0.05.

For this experiment the treatment with FGF2 was selected because it showed the highest blastocyst development among the different growth factors (Table 4). As control, embryos were generated under undefined conditions. A total of 120 expanded embryos were vitrified on day 7 of development, 71 were generated under undefined conditions and 49 under defined conditions (FGF2). The evaluation of embryos at 24 and 48 h post warming showed a similar re-expansion and hatching rate between groups (Figure 1A,B). Specifically, the survival rate assessed after 48 h post warming was higher in the group of embryos cultured under defined (FGF2) conditions (Figure 1C). Quality of embryos, as assessed by the total number of cells, was significantly higher in hatched-warmed embryos generated with FGF2 (199.8 ± 59.6) compared to the undefined FBS condition (146.6 ± 42.8; Figure 1D), while no differences were observed in the proportion of TUNEL-positive cells between groups (Figure 1D).

## 4. Discussion

In this study, we evaluated three different growth factors added individually to IVM and IVC media as a potential replacement for FBS. We were aiming to find one or more growth factors capable of giving similar or better IVP outcomes as compared to the FBS, the most popular additive used to boost the developmental competence in vitro. Our results confirm that gonadotropins are indispensable to induce nuclear maturation and the presence of growth factors is not enough to alleviate the lack of gonadotropins during the induction of in vitro maturation. In addition, by supplementing the embryo culture medium with different growth factors, it was possible to obtain similar developmental outcomes and survival after thawing to the FBS-based IVP system.

Previous studies have shown that EGF stimulates in vitro maturation in different mammalian species, including mouse, rat, human, porcine, and bovine [32,33,34,35]. Here, we have recorded a similar rate of nuclear maturation in TCM-199 containing gonadotropins compared to the same base-medium containing FBS, but maturation rate was lower when the medium was devoid of hormones, even in the presence of growth factors. In line with our data, a previous study indicated that the combination of EGF and gonadotropins during IVM improved the maturation rate and subsequent development in vitro; however, EGF in the absence of gonadotropins did not affect the percentage of oocytes reaching the MII stage [32]. Other studies have also demonstrated that EGF stimulates the expansion of cumulus cells and promotes nuclear maturation of bovine oocytes in vitro [34,36,37,38]. For instance, Cordova et al. (2022) supplemented the maturation medium with EGF (10 ng/ml) and found that blastocyst rates and expression of OCT4 were significantly increased in EGF-treated oocytes [39]. However, no significant differences in nuclear maturation have been reported in other domestic species, such as pigs and sheep [40,41], suggesting species-specific effects.

It has been also previously reported that IGF1 can stimulate oocyte maturation and embryonic development in vitro in several species, including bovine, mouse, pigs, and human [42,43,44,45]. The effect of adding IGF1 in the maturation medium was evaluated earlier by Lorenzo et. al, [38]. These authors observed that supplementation of TCM-199 with IGF1 in the absence of gonadotropins yielded higher nuclear maturation rate compared to a control without growth factors. Although our results show a lower maturation rate in the TCM medium supplemented with IGF1 compared to the TCM containing gonadotropins, the supplementation with IGF1 in the presence of gonadotropins showed similar levels of nuclear maturation and yielded a higher hatching rate. Other authors have indicated positive effects of IGF1 on nuclear and cytoplasmic maturation of oocytes [46,47]. Although, in line with our findings, the addition of IGF1 did not improve nuclear maturation of sheep oocytes in the absence of gonadotropins [41].

Regarding FGF2 supplementation, our data showed that FGF2 induced a similar maturation rate than undefined conditions composed by gonadotropins and FBS. Another study also reported that buffalo oocytes cultured with FGF2 in the presence of gonadotropins displayed a high rate of nuclear maturation [48]. These results agree with previous literature indicating that FGF2 added to regular IVM medium (carrying hormones) improves the degree of cumulus expansion and nuclear maturation [48,49]. Moreover, Barros et al. [50] observed that FGF2 enhanced meiotic resumption, decreased cumulus cell dissociation, and inhibited the apoptosis on cumulus cells. However, Kobayashi et al. [35] reported deficient cleavage rate after supplementation of the MM with FGF2 when the medium was lacking hormones. Therefore, previous literature and our data highlight the importance of gonadotropins for the induction of nuclear maturation of bovine oocytes in vitro.

In Experiment 3, we evaluated the in vitro competence of bovine embryos cultured in chemically defined KSOM medium supplemented with growth factors (EGF, IGF1, or FGF2) individually. The base embryo culture medium (KSOM) supports the in vitro development of IVF, ICSI and somatic cell nuclear transfer (SCNT) bovine embryos [28,51,52,53]. However, a defined embryo culture condition by replacing FBS or BSA with PVA has resulted in poor developmental potential compared to the base medium supplemented with BSA or FBS [54,55]. Our results show similar developmental competence in vitro among all treatments and the control group carrying BSA and FBS, and a higher hatching rate in embryos cultured with IGF1, highlighting the potential role of these growth factors on bovine early development. Similarly, other studies have reported a higher blastocyst rate and of IVF produced embryos cultured under a defined two-step culture medium supplemented with EGF [3]. In agreement with our results, Lott et al., [56] also reported similar development up to the blastocyst stage between undefined and defined embryo culture conditions supplemented with EGF and/or IGF1. A similar result was also described by Wang et al., [57] for bovine SCNT embryos in an embryonic defined medium containing EGF and PVA. In a similar fashion, the addition of IGF-I to the culture medium has also been associated to beneficial effects on the in vitro production of embryos in different species [58,59]. In bovines, the addition of IGF1 increased the morula/blastocyst yield [43,60,61], showed a thermoprotective effect against thermal shock [62], and accelerated the rate of development in vitro [63]. Saeed-Zidane et al. (2019) have indicated that the higher competence and quality of bovine embryos cultured with EGF is associated with changes on DNA methylation and expression of genes involved in the focal adhesion pathway [64].

Nonetheless, studies evaluating the effects of FGF2 in a chemically defined embryo culture medium are scarce. Remarkably, a recent study raised to this cytokine as a potential mediator of maternal and embryo interaction [65]. So far, the literature has shown positive effects on in vitro development when FGF2 is used alone or in combination with other factors. In accordance with our results, the studies of Neira et al., (2010) and Fields et al. (2011) reported positive effects on bovine embryonic development using FGF2 at levels comparable to base medium containing FBS [63,66]. Another study also showed that FGF2, along with TGF-β,1 improved the development of bovine zygotes to the blastocysts stage [67]. Finally, Kumar et al. [49] indicated that FGF2 improved the cleavage rate, but not the yield of morula and blastocysts. These positive effects have been associated with trophic actions of FGF2 on primitive endoderm formation and proliferation during early development [68].

Then, we evaluated the developmental potential of embryos generated in completely defined IVP conditions (during in vitro maturation and embryo culture) using these growth factors individually. The results of this experiment confirm the feasibility to replace FBS and BSA in the maturation and embryo culture media by EGF or FGF2. We observed a similar cleavage rate under defined conditions, except for FGF2, which showed higher cleaving compared to all other groups, including the undefined (FBS/BSA) conditions. Additionally, similar blastocyst rates were observed, except for IGF1, which showed the lowest competence. Only very few studies have assessed the effect of growth factors on the preimplantation development of bovine embryos in completely defined IVP conditions. Previously, Hernandez-Fonseca et al. [69] reported similar in vitro development and pregnancy outcomes after using chemically defined conditions supplemented with IGF1. Additionally, the same group reported that the addition of EGF and IGF1 boosted the developmental competence of bovine embryos [70]. George at al. [71] evaluated a serum-free maturation and embryo culture system supplemented with ITS (insulin, transferrin and selenium). This serum-free embryo culture system improved embryonic development and quality, and the addition of BSA (SOF-ITS-BSA) further improved blastocyst development. Although, the efficiency was inferior to that FBS-supplemented system. 

Finally, we compared the post-thawing survival rate and quality of embryos generated under undefined and defined FGF2 conditions. Accordingly, we found a higher survival rate and total number of cells in hatched blastocysts at 48 h after warming in the FGF2 group. Indeed, a high survival was also observed in the undefined conditions. It is possible that the addition of FGF2 during maturation and embryo culture results in changes in lipid metabolism regulation and increased survivability following cryopreservation. Thus, further research is needed to study the effects of FGF2 on metabolism regulation in bovine early embryogenesis. 

Cell count in the blastocyst is associated with embryo quality, resistance to cryopreservation, and ultimately with pregnancy and deliveries after transfer [14,72,73,74]. The evaluation of the total number of cells showed that the embryos generated under defined conditions showed a quality similar to that obtained in the presence of FBS/BSA. In fact, the quality was similar to previous data found in the literature [75]. In accordance with this, the study of Lee and Fukui [76], indicated improvements on the cell numbers of blastocyst after EGF, although several other reports have described that the numbers of cells were unaffected by the cytokines [18,70,77,78]. Likewise, the addition of IGF1 to embryo culture increased the cell count of bovine blastocysts [60,61,70].

The combination of EGF, IGF1 and/or FGF2 during IVM or embryo culture has also been reported to enhance in vitro embryo production [79,80] and embryo quality via activating mitogen-activated protein kinase pathway [24]. An additive effect was also observed when EGF and IGF1 were used in the maturation medium [38], and embryo culture medium [22]. Stoecklein et al., (2021) supplemented the maturation or embryo culture medium with a cocktail of FGF2, LIF and IGF1 (LFI). These factors improved in vitro maturation, the development competence to the blastocyst stage, and survival following slow freezing, as well as, decreased post-thaw cell apoptosis [25]. Therefore, our data and recent studies are encouraging to address future research on the combined use of EGF, FGF2 and/or IGF1 under defined conditions to further enhance the development potential of embryos generated in these conditions.

## 5. Conclusions

In conclusion, we reported a chemically defined in vitro production system (absence of FBS and BSA during oocyte maturation and embryo culture) based on the use of either EGF or FGF2, which supports the development of bovine embryos of similar quality and post-thawing survival rate to those generated under conditions containing FBS and BSA. These defined embryo production conditions can facilitate pharmacological, toxicological, or metabolic studies, for example, to elucidate nutritional requirements or to identify the molecular pathways that govern the early development in the bovine species. Future studies will aim to assess the effects of the defined conditions on gene expression analyses and/or on the in vivo developmental potential.

## Figures and Tables

**Table 1:** Effect of removing fetal bovine serum and/or hormones from TCM-199-based medium on the in vitro maturation of bovine oocytes.

Treatment	Total Oocytes	Maturation Rate (% ± S.D.)
TCM + H + FBS	334	283 (84 ± 6.0) ^a^
TCM + H	200	157 (78 ± 17) ^a,b^
TCM + EGF	207	128 (62 ± 6.5) ^b,c^
TCM + IGF1	204	106 (51 ± 13) ^c^
TCM + FGF2	215	132 (61 ± 8.4) ^b,c^

Oocytes were randomly allocated to each experimental group and the experiments were replicated 5 times. FBS: fetal bovine serum; TCM: tissue culture medium; H: hormone cocktail (LH, FSH and Estradiol); IGF1: insulin-like growth factor 1, FGF2: fibroblast growth factor 2; EGF: epidermal growth factor. Data followed by different letters in the same column are significantly different (*p* < 0.05).

**Table 2:** Effect of EGF, IGF1 and FGF2 in defined TCM-199 maturation medium on the maturation rate of bovine oocytes.

Treatment	Total Oocytes	Maturation Rate (% ± S.D.)
TCM + H + FBS	221	193 (88 ± 8.0)
TCM + H + EGF	185	167 (90 ± 5.8)
TCM + H + IGF1	190	166 (88 ± 3.6)
TCM + H + FGF2	206	182 (89 ± 5.5)

Oocytes were randomly allocated to each experimental group and the experiments were replicated 7 times. FBS: fetal bovine serum; TCM: tissue culture medium; H: hormone cocktail (LH, FSH and Estradiol); IGF1: insulin-like growth factor 1, FGF2: fibroblast growth factor 2; EGF: epidermal growth factor. No differences were observed between treatments (*p* > 0.05).

**Table 3:** Effect of EGF, IGF1 and FGF2 in defined KSOM embryo culture medium on the in vitro embryonic development of bovine embryos.

Treatment	Total Oocytes	No. Cleaved (%)	No. Blastocysts (%)	Hatched Blastocysts (%)
FBS	243	206 (85)	72 (30)	26 (36) ^a^
EGF	258	205 (79)	61 (24)	35 (57) ^a,b^
IGF1	245	213 (87)	61 (25)	42 (69) ^b^
FGF2	254	217 (85)	66 (26)	38 (58) ^a,b^

Cleavage and blastocyst rates were recorded at 72 and 168 hpf, respectively. Hatched blastocyst rates were recorded at 216 hpf, respectively). Presumptive zygotes were randomly allocated to each experimental group and the experiments were replicated 10 times. FBS: fetal bovine serum; IGF1: insulin-like growth factor 1, FGF2: fibroblast growth factor 2; EGF: epidermal growth factor; KSOM: potassium optimized culture medium. Data followed by different letters in the same column are significantly different (*p* < 0.05).

**Table 4:** Effect of EGF, IGF1 and FGF2 in defined IVP system (in vitro maturation and embryo culture) on developmental potential and total number of cells of bovine embryos.

Treatment	Total Oocytes	No. Cleaved (%)	No. Blastocysts (%)	Total Cells (mean ± S.E.M)
FBS	296	228 (77) ^a^	75 (25) ^a^	165 ± 32
EGF	294	225 (77) ^a^	56 (19) ^a,b^	152 ± 34
IGF1	292	227 (78) ^a,b^	52 (18) ^b^	155 ± 29
FGF2	306	264 (86) ^b^	72 (24) ^a,b^	142 ± 35

Cleavage was recorded at 72 h and blastocyst rates were registered at 168 h (12 replicates). Cell counts were recorded at 168 h in expanded blastocysts (12 blastocysts for each treatment). FBS: fetal bovine serum; IGF1: insulin-like growth factor 1, FGF2: fibroblast growth factor 2; EGF: epidermal growth factor. Data followed by different letters in the same column are significantly different (*p* < 0.05).

## Data Availability

Not applicable.
